# Efficacy and safety of first-line chemoimmunotherapy versus chemotherapy alone for advanced pulmonary lymphoepithelioma-like carcinoma: a systematic review and real-world cohort study

**DOI:** 10.3389/fimmu.2026.1872713

**Published:** 2026-07-01

**Authors:** Feng Chen, Yan Su, Xiandong Zeng, Ming Jiang, Yu Huang, Liuye Pan, Linlin Xiao, Yi rao Qin, Xiangyuan Cen, Jing Bai

**Affiliations:** 1 Department of Respiratory and Critical Care Medicine, The First Affiliated Hospital of Guangxi Medical University, Nanning, Guangxi, China; 2 Department of Hematology, Red Cross Hospital of Yulin City, Yulin, Guangxi, China; 3 Department of Pulmonary and Critical Care Medicine, The First Affiliated Hospital of Guilin Medical University, Guilin, Guangxi, China; 4 Department of Gastrointestinal Oncology, Guangxi Medical University Cancer Hospital, Nanning, Guangxi, China; 5 Department of Radiation Oncology, Affiliated Hospital of Youjiang Medical University For Nationalities, Baise, Guangxi, China

**Keywords:** chemoimmunotherapy, inverse probability of treatment weighting, meta-analysis, pulmonary lymphoepithelioma-like carcinoma, real-world study

## Abstract

**Background:**

Primary pulmonary lymphoepithelioma-like carcinoma (PLELC) is a rare subtype of non-small cell lung cancer characterized by pronounced regional clustering and high PD-L1 expression. Although early studies suggest that first-line immunotherapy combined with chemotherapy has application potential, we still lack large-sample systematic reviews and cross-high-incidence-area real-world validation.

**Methods:**

The electronic databases were systematically searched from inception until January 31, 2026. Studies comparing first-line immunotherapy combined with chemotherapy versus chemotherapy alone in patients with advanced primary pulmonary lymphoepithelioma-like carcinoma (PLELC) were included. To validate the findings, an independent multicenter real-world cohort was concurrently incorporated. The primary endpoints were progression-free survival (PFS) and overall survival (OS), and the secondary endpoints were objective response rate (ORR), disease control rate (DCR), and adverse events (AEs). Stata 15.0 and R software (utilizing inverse probability of treatment weighting [IPTW]) were used to synthesize the meta-analysis and adjust the real-world cohort data, respectively.

**Results:**

The initial meta-analysis (encompassing 606 patients) demonstrates that adding PD-1/PD-L1 inhibitors to chemotherapy significantly prolongs both progression-free survival (PFS) and overall survival (OS) without elevating the risk of severe adverse events. Within the independent multicenter cohort from Guangxi, the combination therapy group continues to display a profound PFS benefit after IPTW strictly balances the baseline disease burden (18.0 vs. 6.5 months, HR = 0.17, P < 0.001). OS in this cohort exhibits a protective trend (HR = 0.85); however, incorporating this real-world effect size into the global meta-model yields updated pooled data that further reinforces its robust, cross-regional survival advantage (PFS HR = 0.42; OS HR = 0.51). Multivariate prognostic analysis identifies baseline liver metastasis as an independent risk factor for reduced OS, whereas PD-L1 positivity significantly correlates with improved PFS.

**Conclusion:**

This integrated systematic review and multicenter real-world cohort study suggests the superior efficacy and safety of first-line chemoimmunotherapy over chemotherapy alone, providing evidence-based support for the management of advanced PPLELC.

## 1 Introduction

Primary pulmonary lymphoepithelioma-like carcinoma (PPLELC) is a distinct entity of non-small cell lung cancer (NSCLC) (1) that is highly associated with Epstein-Barr virus (EBV) infection (2–4). It is classified as a distinct subtype of pulmonary squamous cell carcinoma by the 2021 WHO Classification of Thoracic Tumors (5). In terms of both histopathological features and epidemiological profile, this disease shares a profound overlap with nasopharyngeal carcinoma, with its endemicity primarily concentrated in southern China (particularly the Guangdong and Guangxi regions) and Southeast Asia (6, 7). The clinical and molecular landscape of PPLELC presents a significant therapeutic challenge: patients are generally younger non-smokers who lack conventional targets like ALK or EGFR (8–10). Consequently, while initial platinum-based chemotherapy may induce responses, these are usually brief, resulting in unfavorable long-term outcomes (7, 8, 11). In stark contrast to its genomic silence, PPLELC exhibits a highly immunogenic, “hot” microenvironment (7, 12). The pronounced infiltration of immune cells and overwhelmingly positive PD-L1 expression establish a robust theoretical foundation for the use of immune checkpoint inhibitors (ICIs) (13).

Recent retrospective cohorts have investigated the efficacy of first-line chemoimmunotherapy in advanced PPLELC, indicating potential survival benefits superior to those of chemotherapy alone (14, 15).Nevertheless, the extreme rarity of PPLELC has precluded the execution of large-scale systematic reviews, keeping the current level of clinical evidence relatively low (16). Furthermore, existing research is overwhelmingly restricted to Guangdong province (17), leaving a critical knowledge void regarding other major endemic epicenters like the Guangxi Zhuang Autonomous Region. Additionally, anti-angiogenic agents are known to reverse microenvironmental hypoxia and favorably modulate immune cell infiltration—a synergy with ICIs that is well-established across multiple solid tumors (18, 19). The therapeutic efficacy and safety profile between ICIs and anti-angiogenic therapies have not yet been systematically evaluated in PPLELC.

Addressing these limitations, we conducted the first meta-analysis evaluating the efficacy and safety of first-line chemoimmunotherapy versus chemotherapy alone in advanced PPLELC. We independently validated these findings using a multicenter, real-world cohort from Guangxi and incorporated the effect sizes from this cohort into the meta-analysis model to update the global chain of evidence. Building upon this, we further explored the preliminary efficacy and tolerability of ICIs combined with anti-angiogenic targeted therapy (± chemotherapy) in a real-world setting. Ultimately, this study aims to enrich the evidence-based medical framework for advanced PPLELC, providing multidimensional insights to optimize individualized, first-line clinical decision-making.

## 2 Materials and methods

### 2.1 Systematic review and meta-analysis

The current systematic review and meta-analysis complied with the Preferred Reporting Items for Systematic Reviews and Meta-Analyses (PRISMA) statement (20). Prior to initiation, the review protocol was registered on PROSPERO (identifier: CRD420251232602). Two authors (F.C and Y.S) conducted the literature screening and data collection independently. Disagreements were resolved by discussing with a third reviewer (J.B) to reach a consensus.

#### 2.1.1 Search strategy

We systematically searched PubMed, Embase, Web of Science, and major Chinese biomedical databases, including China National Knowledge Infrastructure (CNKI), Wanfang Data, VIP Database, and the Chinese Biomedical Literature Database (CBM), for relevant records published from database inception to January 31, 2026. No language restriction was applied.The search strategy combined controlled vocabulary terms, including Medical Subject Headings (MeSH) where applicable, and free-text terms. Search terms focused on the disease of interest, including “pulmonary lymphoepithelioma-like carcinoma,” “primary pulmonary lymphoepithelioma-like carcinoma,” “PPLELC,” and their Chinese equivalents, as well as treatment-related terms including “chemoimmunotherapy,” “immunotherapy,” “PD-1/PD-L1 inhibitors,” “CTLA-4,” “pembrolizumab,” “nivolumab,” “chemotherapy,” and “drug therapy.” The complete English and Chinese search strategies are provided in **Supplementary Table 2**.

#### 2.1.2 Eligibility criteria and endpoints

Eligible studies met the following criteria (1): enrolled patients had a pathological diagnosis of stage IIIB–IV PPLELC (2); the study compared first-line chemoimmunotherapy against chemotherapy alone; (3) reported at least one survival (overall survival [OS] or progression-free survival [PFS]) or tumor response outcome (objective response rate [ORR] or disease control rate [DCR]).During data extraction, we strictly verified that all included cohorts utilized uniform outcome definitions. Specifically, tumor responses (ORR and DCR) and disease progression were uniformly assessed according to the RECIST v1.1 criteria. Based on this, PFS was calculated from the start of treatment to the first documented RECIST-defined progression or death, and OS was strictly defined as the time to all-cause mortality. ORR represented the percentage of evaluated patients showing either a complete (CR) or partial response (PR). DCR included CR, PR, and stable disease (SD) rates. We excluded animal models, reviews, case reports, and articles with unextractable data. When different publications reported on overlapping patient populations from the same institution, only the dataset with the largest sample size or longest follow-up was included to prevent duplicated data.

#### 2.1.3 Data extraction and quality assessment

Information extracted from each study included baseline characteristics (e.g., first author, year of publication, sample size, disease stage), hazard ratios (HRs) with 95% confidence intervals (CIs) for OS and PFS, and relative risks (RRs) with 95% CIs for ORR, DCR, and adverse events (AEs). The methodological quality of all included retrospective studies was assessed using the Newcastle-Ottawa Scale (NOS). This scale evaluates studies based on patient selection, group comparability, and outcome assessment. Studies receiving a score > 6 (out of a maximum 9) were classified as high quality (21).

#### 2.1.4 Data extraction and quality assessment

Statistical pooling was performed using Stata version 15.0. If HRs and their corresponding 95% CIs were not explicitly provided in the original texts, we estimated them from the presented Kaplan-Meier curves applying the Tierney method (22). We used the *I*² index and Cochran’s Q test to assess between-study heterogeneity. A fixed-effects model was chosen for analysis if heterogeneity was low (*P* > 0.10 and *I*² < 50%) (23, 24); otherwise, a random-effects approach was adopted. Radial and L’Abbé plots were generated to visualize potential clinical heterogeneity. Furthermore, a leave-one-out sensitivity analysis was carried out to test the stability of the pooled results. Potential publication bias was evaluated visually through funnel plots and quantitatively assessed using Begg’s and Egger’s tests (25).

### 2.2 Real-world validation cohort

#### 2.2.1 Clinical data sources and patient selection

We gathered retrospective data on newly diagnosed advanced PPLELC patients treated across five tertiary hospitals in Guangxi (First Affiliated Hospital of Guangxi Medical University, Guangxi Medical University Cancer Hospital,First Affiliated Hospital of Guilin Medical University, Red Cross Hospital of Yulin City, Affiliated Hospital of Youjiang Medical University For Nationalities) from January 1, 2019, to December 13, 2025. The institutional review boards (IRBs) at each participating site approved the study protocol. Patient consent was waived due to the retrospective nature of the analysis, and all procedures complied with the Declaration of Helsinki.To be included, patients had to meet the following criteria: (1) initial pathological or cytological diagnosis of PPLELC; (2) positive EBER *in situ* hybridization results, alongside clinical imaging that ruled out pulmonary metastasis originating from nasopharyngeal cancer; (3) stage IIIB to IV disease based on the 8th edition of the AJCC TNM classification, without surgical indications; (4) availability of complete baseline and follow-up records; (5) confirmed administration of first-line platinum doublet chemotherapy or chemoimmunotherapy (involving PD-1/PD-L1 blockade).We assigned patients to either the chemotherapy (Chemo) or chemoimmunotherapy (IO+Chemo) cohort according to their initial treatment records. Due to the limited sample size within each specific pharmacological subgroup and to prevent model overfitting during subsequent propensity score weighting and multivariable analyses, these interventions were strictly evaluated at the class level (i.e., platinum-based doublets and PD-1/PD-L1 inhibitors). We also formed a separate, exploratory subgroup (IO + Anti-angio ± Chemo).

#### 2.2.2 Efficacy evaluation and follow-up

Patients underwent imaging scans every 6 to 8 weeks during the follow-up period. Two independent researchers assessed objective tumor responses in a double-blind fashion applying the RECIST v1.1 guidelines. PFS served as the primary endpoint. Secondary endpoints were OS, ORR, and DCR.

### 2.3 Propensity score weighting and survival model construction

#### 2.3.1 Inverse probability of treatment weighting

We applied inverse probability of treatment weighting (IPTW) to balance baseline patient characteristics and control for observable confounders between the two treatment arms. For each patient, the propensity score (PS) for receiving chemoimmunotherapy was estimated via a multivariable logistic regression model. The covariates included in this model were factors known to drive clinical treatment decisions and affect survival: age, sex, smoking history, ECOG performance status, clinical TNM stage, presence of liver or bone metastases, and PD-L1 expression levels.To check the positivity assumption, we plotted the PS distributions using kernel density estimation, which confirmed adequate common support between the groups. We then computed stabilized weights and trimmed the top and bottom 1% of the weight distribution to prevent extreme values from inflating the variance (26). Covariate balance post-weighting was assessed by calculating the standardized mean difference (SMD), with a value below 0.1 indicating optimal balance. A Love plot was generated to visualize the SMD shifts before and after adjustment (26).

#### 2.3.2 Survival model analysis

All statistical analyses and plotting for the real-world data were performed in R (version 4.4.1) using the WeightIt, survey, and survival packages. In the IPTW-adjusted cohort, we calculated the median OS and PFS via the weighted Kaplan-Meier method and compared the survival curves using a weighted log-rank test. After verifying the proportional hazards (PH) assumption, we fitted a weighted Cox proportional hazards regression model to determine the effect sizes. Because the weighting process introduces non-independence among sample observations, we incorporated robust standard errors into the model. Robust Wald tests were then used to compute the adjusted hazard ratios (HRs), 95% CIs, and P-values.

#### 2.3.3 Exploratory subgroup analysis

Only 12 patients received an anti-angiogenic combination regimen. Due to this limited sample size and the clinical heterogeneity of the treatments (involving mixed doublet or triplet regimens across different treatment lines), we only performed descriptive analyses for this subgroup to avoid unreliable statistical inferences. We used a swimmer plot to map the individual treatment durations and clinical responses.

### 2.4 Evidence integration and heterogeneity analysis

To validate the treatment efficacy across different endemic populations, we extracted the adjusted HRs and 95% CIs from the IPTW-matched Guangxi cohort and added them directly into our initial meta-analysis model. This secondary pooling step allowed us to test the stability of the overall effect sizes after incorporating the new real-world data. It also enabled us to explore potential regional heterogeneity in clinical outcomes between the Guangdong and Guangxi cohorts, generating more broadly applicable evidence for managing advanced PPLELC.

## 3 Results

### 3.1 Meta-analysis of previous evidence

#### 3.1.1 Literature search and baseline characteristics of included studies

**Figure 1:**
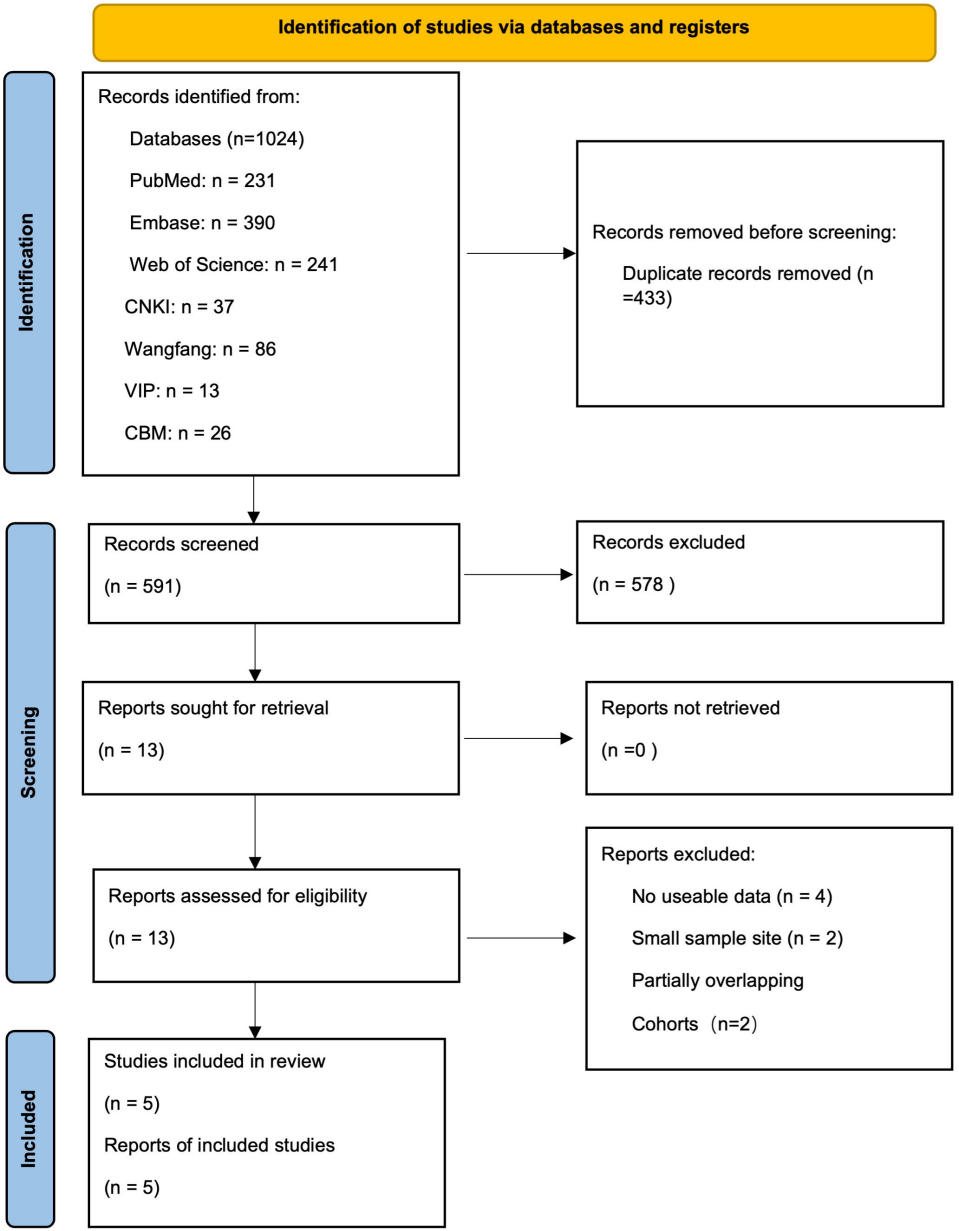
PRISMA flow diagram of study selection.

We outlined the literature search and selection process according to the PRISMA flow diagram (**Figure 1**). Initial searches across the PubMed, Embase, and Web of Science,CNKI, Wanfang, VIP, and CBM databases yielded 1024 records. After removing duplicates and screening the remaining records step-by-step, we included 5 publications representing 4 independent retrospective cohorts (one publication was an updated survival analysis of an existing cohort) (17, 27–30). **Table 1** summarizes the baseline characteristics, extracted survival data, and quality assessment of these included studies. Published between 2022 and 2026, these studies encompassed a total of 606 patients with advanced PPLELC. Patients in the intervention arms received first-line PD-1/PD-L1 inhibitors combined with platinum doublet chemotherapy, whereas the control groups received platinum doublet chemotherapy alone. Based on the Newcastle-Ottawa Scale (NOS) evaluation, all included cohorts demonstrated high methodological quality (**Supplementary Table 2**).

**Table 1:** Main characteristics of the included studies in the meta-analysis.

Study	Study design	No. of patients (Chemo/IO)	Clinical stage	Median survival, mos (Chemo/IO)		Hazard ratio (95% CI)		Survival outcomes	Quality score
				PFS	OS	PFS	OS		
Hou 2025	Retrospective	116/106	IV	NA	NA	0.485(0.375–0.658)	0.485(0.375–0.658)	PFS, OS	6
Chen 2025	Retrospective	88/102	IIIB-IV	7.2(6.4 - 8.5) / 16.5(11.2 - 21.7)	44.4 / NR	0.5(0.26–0.96)	NA	PFS, OS	8
Xiao 2022	Retrospective	49/12	IIIB-IV	6.9(2.3-NA) / 11.8(6-NA)	NA	0.38(0.18-0.78)	NA	PFS	7
Zhang 2022	Retrospective	78/55	IIIB-IV	7.7(6.8-8.6) / 12.8(5.2-20.4)	35.7(26.7-44.8) / NR	0.48(0.31-0.74)	0.47(0.20-1.07)	PFS, OS	8
Tang 2026	Retrospective	78/55	IIIB-IV	7.6( 6.8–8.5) / 15.7( 8.1–23.3)	25.6 / NR	0.45 (0.30–0.67)	0.48 (0.30–0.77)	PFS, OS	9

Chemo, chemotherapy; IO, immunotherapy; PFS, progression-free survival; OS, overall survival; HR, hazard ratio; CI, confidence interval; NOS, Newcastle-Ottawa Scale; NA, not available; NR, not reached.Note: All included studies were conducted in China.

#### 3.1.2 Pooled analysis of survival outcomes, tumor response, and safety

**Figure 2:**
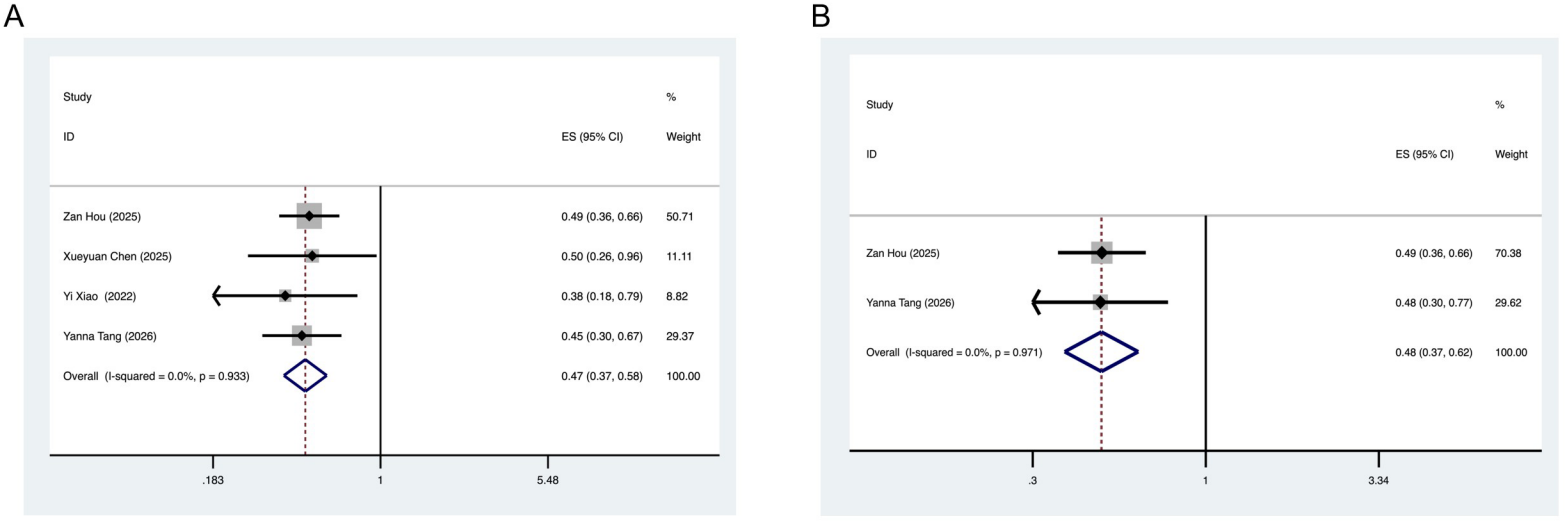
Forest plot of hazard ratios for PFS**(A)** and OS **(B)** in patients with PLELC receiving first-line chemo-immunotherapy versus chemotherapy alone. PLELC, pulmonary lymphoepithelioma like carcinoma; PFS, Progression-Free Survival;OS, overall survival.

Because we detected no significant statistical heterogeneity across the primary survival endpoints, we applied a fixed-effects model for data pooling (**Table 2**). Survival Outcomes: Compared with chemotherapy alone, first-line chemoimmunotherapy significantly improved progression-free survival (PFS), reducing the risk of disease progression by 53% (pooled HR = 0.47, 95% CI: 0.37–0.58, P < 0.001; I² = 0%). Overall survival (OS) also improved significantly, with a 52% reduction in mortality risk for the combination group (pooled HR = 0.48, 95% CI: 0.37–0.62, P < 0.001; I² = 0%) (**Figure 2**, **Table 2**).

**Table 2:** Pooled effect sizes of first-line immunotherapy combined with chemotherapy versus chemotherapy alone (Initial and Updated meta-analysis).

Outcomes	Analysis phase	No. of studies	Effect size (95% CI) a	P value	Heterogeneity (I², P value)
PFS	Initial	4	0.47 (0.37–0.58)	< 0.001	I² = 0%, P = 0.933
	Updated	5	0.42 (0.35–0.52)	< 0.001	I² = 47%, P = 0.110
OS	Initial	2	0.48 (0.37–0.62)	< 0.001	I² = 0%, P = 0.971
	Updated	3	0.51 (0.40–0.65)	< 0.001	I² = 0%, P = 0.463
ORR	Initial	2	1.86 (1.50–2.32)	< 0.001	I² = 13.9%, P = 0.281
	Updated	3	1.81 (1.48–2.23)	< 0.001	I² = 0%, P = 0.461
DCR	Initial	3	1.11 (1.04–1.17)	0.001	I² = 0%, P = 0.434
	Updated	4	1.10 (1.04–1.16)	0.001	I² = 0%, P = 0.546
Grade ≥ 3 AEs	Initial	2	0.94 (0.74–1.19)	0.599	I² = 0%, P = 0.600

AE, adverse event; CI, confidence interval; DCR, disease control rate; ORR, objective response rate; OS, overall survival; PFS, progression-free survival.

Note: A fixed-effects model was applied for all pooled analyses due to low statistical heterogeneity (I² < 50%).

**Figure 3:**
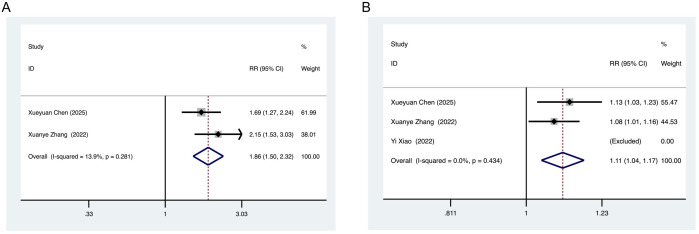
Forest plot of relative risk for ORR **(A)** and DCR **(B)** in patients with PLELC receiving first-line chemo-immunotherapy versus chemotherapy alone. PLELC, pulmonary lymphoepithelioma like carcinoma; ORR, Objective Response Rate; DCR, Disease Control Rate.

**Figure 4:**
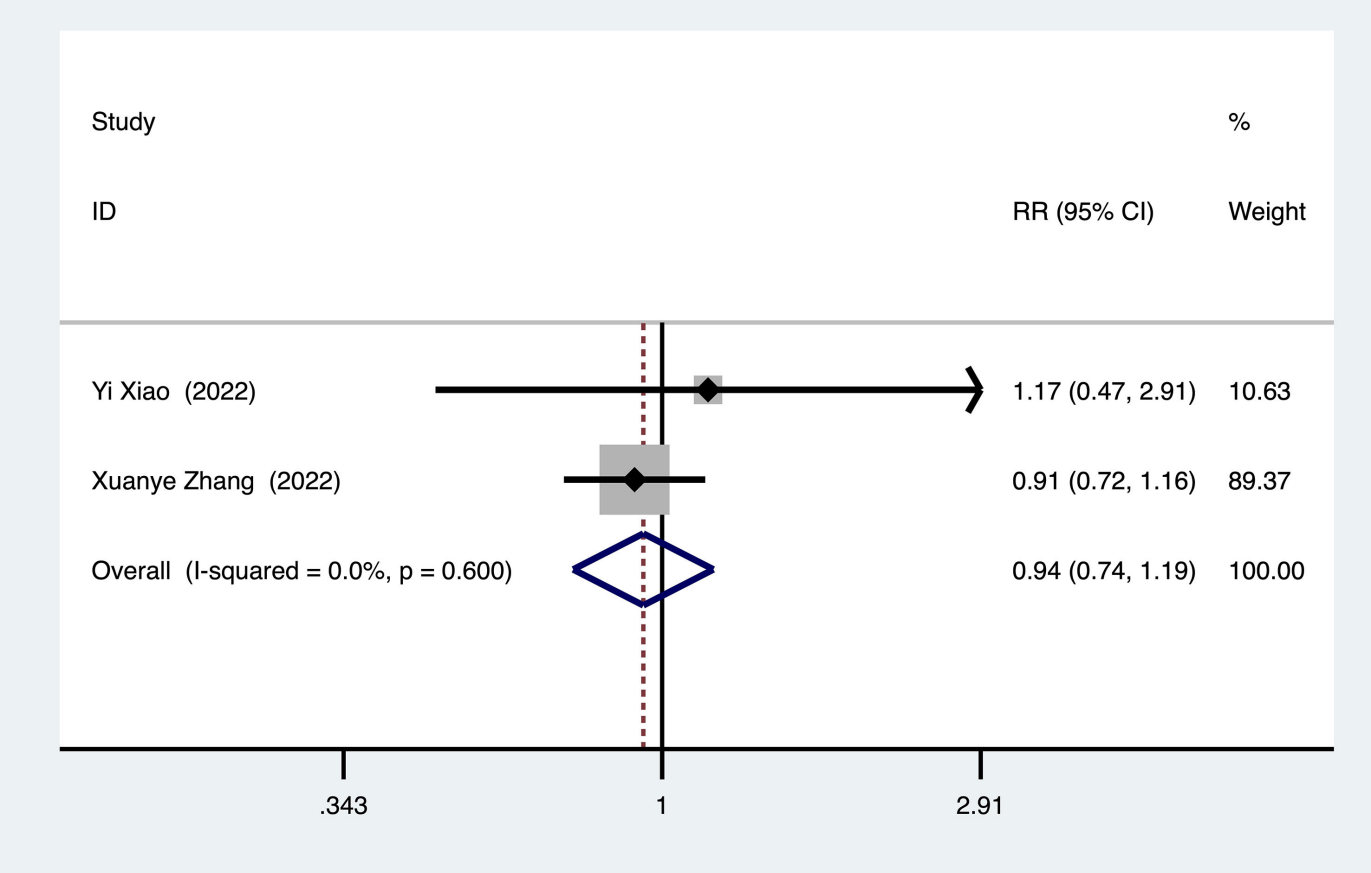
Forest plot of relative risk for grade 3/4 TRAE in patients with PLELC receiving first-line chemo-immunotherapy versus chemotherapy alone. PLELC pulmonary lymphoepithelioma-like carcinoma; TRAE, Treatment-Related Adverse Event.

Tumor Response and Safety: For tumor response, the initial pooled analysis of the objective response rate (ORR) revealed high heterogeneity (I² = 78.3%) (**Supplementary Figure 1**). Visual inspections using L’Abbé and radial plots, coupled with a leave-one-out sensitivity analysis (31), identified one small-sample study as the primary source of this heterogeneity (**Supplementary Figure 2**-**4**). Excluding this study markedly reduced the heterogeneity (I² = 13.9%) without altering the direction of the overall effect. The adjusted pooled ORR remained significantly higher in the chemoimmunotherapy arm (pooled RR = 1.86, 95% CI: 1.50–2.32, P < 0.001). The disease control rate (DCR) also favored the combination group (pooled RR = 1.11, 95% CI: 1.04–1.17, P = 0.001; I² = 0%). Regarding tolerability, the risk of grade ≥ 3 adverse events (AEs) did not differ significantly between the two arms (pooled RR = 0.94, 95% CI: 0.74–1.19, P = 0.599; I² = 0%), indicating that the addition of first-line immunotherapy did not significantly increase the severe toxicity burden (**Figures 3**, **4**, **Table 2**).

Sensitivity analyses confirmed the robustness of these findings, as the pooled effect sizes remained stable when omitting individual studies sequentially (**Supplementary Figure 5**). Funnel plots and Begg’s tests were constructed to assess potential publication bias, Begg’s tests showed no statistical significance (all P > 0.05, **Supplementary Figure 6**).However, given the small number of included studies, the funnel plot and Begg’s tests lack sufficient statistical power to definitively rule out publication bias; these results should therefore be interpreted with caution.

### 3.2 Baseline characteristics of the guangxi real-world cohort

We initially screened 276 patients with advanced PPLELC from five major medical centers in Guangxi.Applying strict eligibility criteria yielded a final real-world cohort of 71 patients. These patients were categorized into a chemotherapy alone (Chemo) arm (n = 17) and a chemoimmunotherapy (IO+Chemo) arm (n = 48). The detailed descriptive distribution of the specific platinum backbones, non-platinum partners, and PD-1/PD-L1 inhibitors administered across these two main cohorts is summarized in **Supplementary Table 3**. Additionally, an exploratory anti-angiogenic combination subgroup of 12 patients was established (6 of whom overlapped with the primary cohorts due to concurrent regimens, and 6 were independently enrolled).Within the unadjusted raw cohort, the IO+Chemo group presented with a noticeably heavier baseline disease burden compared to the Chemo group. Specifically, they had higher proportions of liver metastases (29.2% vs. 0%), and bone metastases (33.3% vs. 5.9%). These imbalances reflect routine clinical practice, where physicians tend to prescribe combination immunotherapy for patients harboring high-risk prognostic features (**Table 3**).We built a propensity score (PS) model utilizing predefined prognostic covariates to adjust for these baseline confounders. A kernel density plot (**Supplementary Figure 7**) confirmed adequate common support between the two arms, primarily within the 0.1 to 0.6 PS range, fulfilling the core assumption for weighting. This common support held true even though the IO+Chemo scores skewed right with a long tail in the higher ranges.A Love plot (**Supplementary Figure 8**) mapped the covariate shifts before and after adjustment. The IPTW procedure successfully balanced key baseline characteristics—such as age, sex, ECOG performance status, TNM stage, and metastatic sites—yielding standardized mean differences (SMDs) below 0.1 across these variables. While minor residual imbalances persisted in a few metastasis-related covariates—driven by the extremely low baseline metastatic rate in the chemotherapy arm—the resulting pseudo-randomized cohort provided a sound statistical framework. This allowed us to accurately assess the survival benefits of chemoimmunotherapy despite the group’s inherently higher baseline risk (**Table 3**).

**Table 3:** Baseline characteristics of patients before and after IPTW adjustment.

Characteristics	Unweighted cohort				IPTW-Adjusted cohort			
	Chemotherapy	Immuno-chemo	P value	SMD	Chemotherapy	Immuno-chemo	P value	SMD
n	17	48			17.5	47.7		
Age_Group			0.322	0.364			0.87	0.042
<=60	12 (70.6)	41 (85.4)			14.7 (84.2)	39.4 (82.6)		
>60	5 (29.4)	7 (14.6)			2.8 (15.8)	8.3 (17.4)		
Gender			0.538	0.258			0.955	0.017
Female	11 (64.7)	25 (52.1)			9.5 (54.4)	26.3 (55.2)		
Male	6 (35.3)	23 (47.9)			8.0 (45.6)	21.4 (44.8)		
Smoking			0.493	0.374			0.255	0.486
never	15 (88.2)	36 (75.0)			16.3 (93.0)	36.4 (76.3)		
former	0 (0.0)	1 (2.1)			0.0 (0.0)	1.0 (2.0)		
current	2 (11.8)	11 (22.9)			1.2 (7.0)	10.3 (21.6)		
Stage_Group			0.389	0.326			0.809	0.064
Stage III	6 (35.3)	10 (20.8)			3.6 (20.7)	11.1 (23.3)		
Stage IV	11 (64.7)	38 (79.2)			13.9 (79.3)	36.6 (76.7)		
ECOG_PS			0.97	0.295			0.386	0.305
0	0 (0.0)	2 (4.2)			0.0 (0.0)	2.1 (4.5)		
1	17 (100.0)	46 (95.8)			17.5 (100.0)	45.6 (95.5)		
PDL1_TPS			0.297	0.468			0.527	0.356
<1%	3 (17.6)	5 (10.4)			2.8 (15.9)	5.3 (11.0)		
>=1%	3 (17.6)	18 (37.5)			3.9 (22.0)	18.1 (37.9)		
Unknown	11 (64.7)	25 (52.1)			10.8 (62.0)	24.3 (51.1)		
Met_Liver			0.03	0.907			0.012	0.904
No	17 (100.0)	34 (70.8)			17.5 (100.0)	33.8 (71.0)		
Yes	0 (0.0)	14 (29.2)			0.0 (0.0)	13.8 (29.0)		
Met_Bone			0.058	0.737			0.152	0.572
No	16 (94.1)	32 (66.7)			15.8 (90.3)	32.4 (68.0)		
Yes	1 (5.9)	16 (33.3)			1.7 (9.7)	15.2 (32.0)		

IPTW, inverse probability of treatment weighting; SMD, standardized mean difference; ECOG PS, Eastern Cooperative Oncology Group performance status; PD-L1, programmed cell death ligand 1. SMD < 0.1 indicates a negligible different;TPS, tumor proportion score.

### 3.3 Survival analysis and prognostic exploration of the real-world cohort

#### 3.3.1 Survival outcomes in the IPTW-adjusted cohort

**Figure 5:**
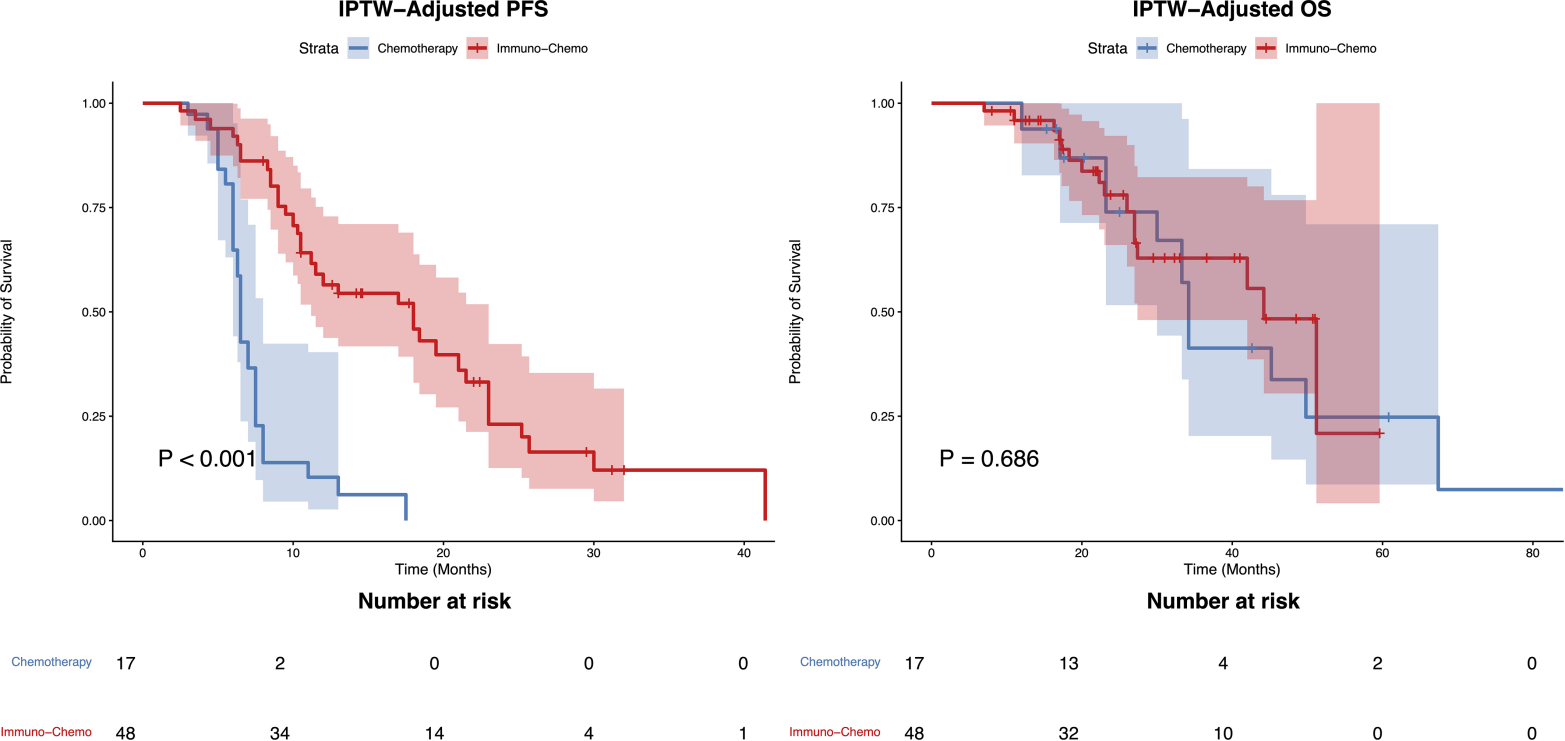
Kaplan-Meier estimates of survival outcomes in the real-world PLELC cohort after inverse probability of treatment weighting (IPTW) adjustment. Progression-free survival (PFS). Overall survival (OS). Chemo, chemotherapy; CI, confidence interval; HR, hazard ratio; IO, immunotherapy; IPTW, inverse probability of treatment weighting; OS, overall survival; PFS, progression-free survival;.

At a median follow-up of 32 months, the inverse probability of treatment weighting (IPTW)-adjusted Kaplan-Meier analysis revealed a substantial progression-free survival (PFS) benefit for patients receiving first-line chemoimmunotherapy. The weighted median PFS reached 18.0 months (95% CI: 11.0–23.0) in the IO+Chemo arm, exceeding the 6.5 months (95% CI: 6.0–8.0) observed in the chemotherapy alone arm. A robust Wald test derived from the weighted Cox proportional hazards model confirmed an 82% reduction in the risk of disease progression (adjusted HR = 0.17, 95% CI: 0.09–0.34, P < 0.001) (**Figure 5**).Regarding overall survival (OS), the weighted median OS was 44.2 months (95% CI: 27.0–NR) for the combination group and 34.2 months (95% CI: 30.0–NR) for the chemotherapy group. Although the chemoimmunotherapy regimen indicated a long-term protective trend with an 18% numerical decrease in mortality risk (adjusted HR = 0.85, 95% CI: 0.39–1.86), this difference did not reach statistical significance (P = 0.686) (**Figure 5**).The follow-up compliance in our cohort exceeded 80%. Early dropouts due to severe toxicity or rapid deterioration were rare; thus, the vast majority of censored OS events were purely administrative (i.e., patients remaining alive at data cutoff).

#### 3.3.2 Univariable and multivariable cox regression analyses

The weighted multivariable Cox regression model identified several key clinical determinants of survival. Receiving first-line chemoimmunotherapy emerged as a strong independent protective factor for PFS (adjusted HR = 0.12, 95% CI: 0.06–0.23, P < 0.001), as did positive baseline PD-L1 expression (P < 0.05). Conversely, the presence of baseline liver metastasis acted as a significant independent risk factor, driving both earlier disease progression (PFS adjusted HR = 3.16, 95% CI: 1.25–7.99, P = 0.015) and a notably shortened overall survival (OS adjusted HR = 4.96, 95% CI: 1.63–15.03, P = 0.005) (**Tables 4A**, **4B**).

**Table 4A:** Univariate and multivariate cox regression analysis for progression-free survival (PFS).

Variable	Univariate analysis		Multivariate analysis (IPTW)	
	HR (95% CI)	P value	HR (95% CI)	P value
Treatment regimen				
Chemotherapy	Reference	-	Reference	-
Immuno-chemotherapy	0.19 (0.10-0.37)	< 0.001	0.12 (0.06-0.23)	< 0.001
Age				
<= 60 years	Reference	-		
> 60 years	1.44 (0.73-2.81)	0.292		
Gender				
Female	Reference	-		
Male	0.74 (0.41-1.31)	0.295		
Smoking status				
Never	Reference	-	Reference	-
Former	0.46 (0.06-3.40)	0.444	0.59 (0.27-1.27)	0.177
Current	1.55 (0.81-2.98)	0.190	1.62 (0.75-3.49)	0.223
Clinical stage				
Stage III	Reference	-	Reference	-
Stage IV	0.84 (0.46-1.55)	0.585	1.08 (0.56-2.08)	0.822
ECOG PS				
0	Reference	-		
1	NE	NE		
PD-L1 expression				
< 1%	Reference	-	Reference	-
>= 1%	0.44 (0.18-1.10)	0.078	0.46 (0.19-1.13)	0.092
Unknown	0.69 (0.30-1.59)	0.383	0.73 (0.36-1.49)	0.390
Liver metastasis				
No	Reference	-	Reference	-
Yes	1.43 (0.73-2.80)	0.298	3.16 (1.25-7.99)	0.015
Bone metastasis				
No	Reference	-		
Yes	1.00 (0.53-1.89)	0.995		

IPTW, inverse probability of treatment weighting; SMD, standardized mean difference; ECOG PS, Eastern Cooperative Oncology Group performance status; PD-L1, programmed cell death ligand 1. SMD < 0.1 indicates a negligible different;TPS, tumor proportion score.Note: NE, not estimable due to no events in the subgroup.

**Table 4B:** Univariate and multivariate cox regression analysis for overall survival (OS).

Variable	Univariate analysis		Multivariate analysis (IPTW)	
	HR (95% CI)	P value	HR (95% CI)	P value
Treatment regimen				
Chemotherapy	Reference	-	Reference	-
Immuno-chemotherapy	1.01 (0.43-2.40)	0.976	0.46 (0.17-1.26)	0.130
Age				
<= 60 years	Reference	-		
> 60 years	0.49 (0.17-1.48)	0.207		
Gender				
Female	Reference	-		
Male	1.05 (0.47-2.37)	0.897		
Smoking status				
Never	Reference	-	Reference	-
Former	1.64 (0.21-12.61)	0.633	3.58 (1.00-12.73)	0.049
Current	1.60 (0.63-4.08)	0.321	1.52 (0.51-4.55)	0.452
Clinical stage				
Stage III	Reference	-	Reference	-
Stage IV	2.14 (0.83-5.51)	0.117	2.01 (0.65-6.19)	0.224
ECOG PS				
0	Reference	-		
1	NE	NE		
PD-L1 expression				
< 1%	Reference	-	Reference	-
>= 1%	0.65 (0.19-2.27)	0.499	0.69 (0.14-3.29)	0.640
Unknown	0.67 (0.21-2.13)	0.503	1.08 (0.25-4.60)	0.916
Liver metastasis				
No	Reference	-	Reference	-
Yes	3.89 (1.53-9.85)	0.004	4.96 (1.63-15.03)	0.005
Bone metastasis				
No	Reference	-		
Yes	2.18 (0.93-5.15)	0.075		

IPTW, inverse probability of treatment weighting; SMD, standardized mean difference; ECOG PS, Eastern Cooperative Oncology Group performance status; PD-L1, programmed cell death ligand 1. SMD < 0.1 indicates a negligible different;TPS, tumor proportion score.Note: NE, not estimable due to no events in the subgroup.

#### 3.3.3 PD-L1 subgroup analysis

**Figure 6:**
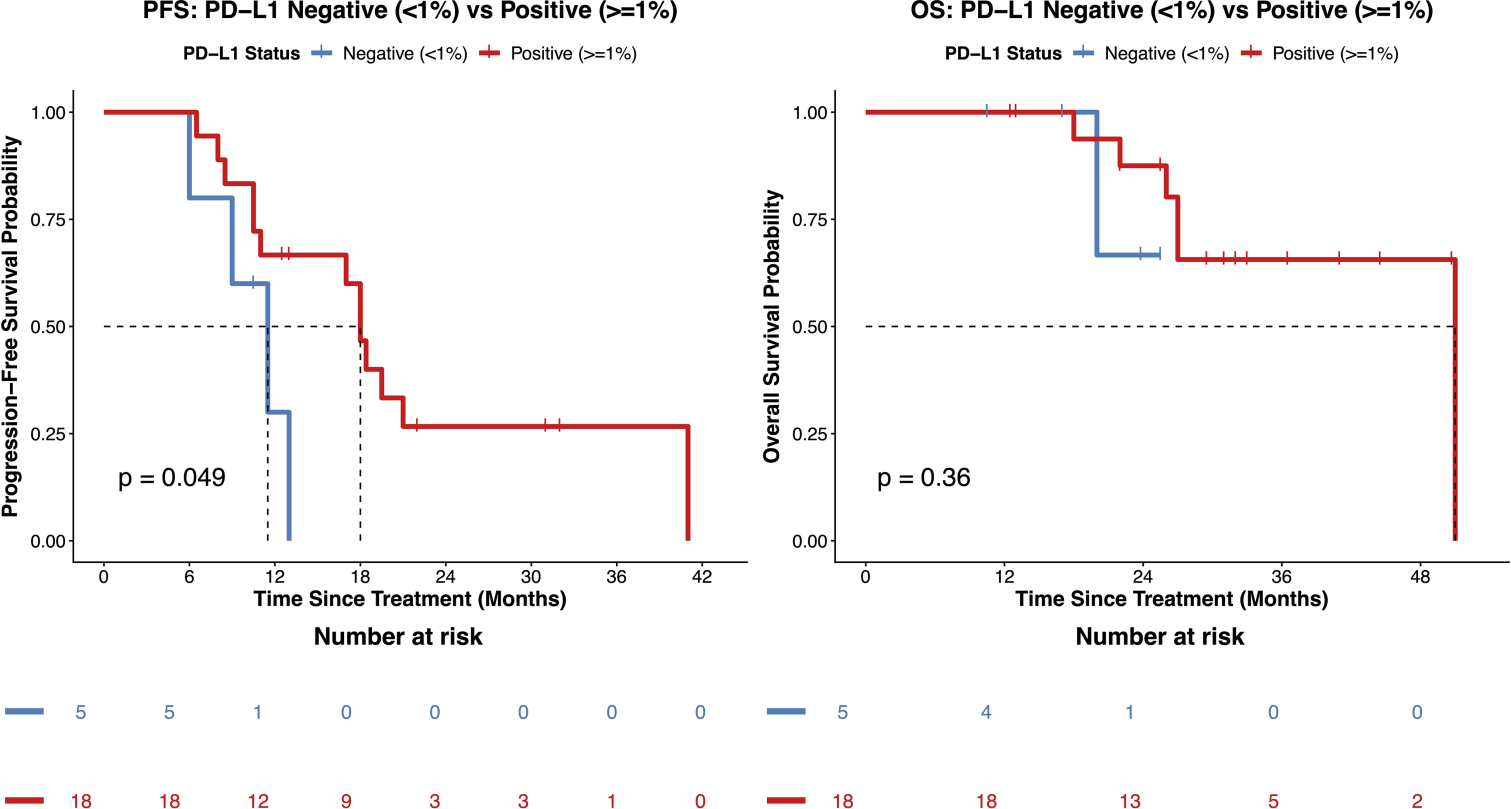
Kaplan-Meier analysis of survival outcomes stratified by baseline PD-L1 expression status in patients receiving first-line immunotherapy. Progression-free survival (PFS). Overall survival (OS). No significant difference in OS was observed between the PD-L1 positive and negative subgroups (median OS, 51.0 months vs. not reached, P = 0.360). OS, overall survival; PFS, progression-free survival;.

We evaluated the predictive value of PD-L1 expression in a subset of 23 patients with available baseline testing data (18 with PD-L1 ≥ 1% and 5 with PD-L1 < 1%). Kaplan-Meier survival estimates demonstrated that patients harboring PD-L1-positive tumors experienced a significantly longer PFS than those with PD-L1-negative tumors (median PFS: 18.0 vs. 11.5 months, P = 0.049).For OS, the median was 51.0 months in the PD-L1-positive group, while the median OS for the negative group remained unreached due to a low number of death events (P = 0.36). These exploratory findings suggest that baseline PD-L1 positivity may serve as a potential biomarker predicting PFS benefits from first-line chemoimmunotherapy. However, the extremely small sample size of the PD-L1-negative cohort (n = 5) severely limits the statistical power of this analysis, meaning the robustness of this survival difference requires further validation in larger cohorts (**Figure 6**).

### 3.4 Updated meta-analysis integrating regional real-world evidence

To test the clinical generalizability of the combination regimen across different geographic populations, we treated the IPTW-adjusted effect sizes from our Guangxi real-world cohort as an independent dataset and incorporated them into the global meta-analysis model (**Table 2**).

#### 3.4.1 Updated survival evidence (PFS and OS)

**Figure 7:**
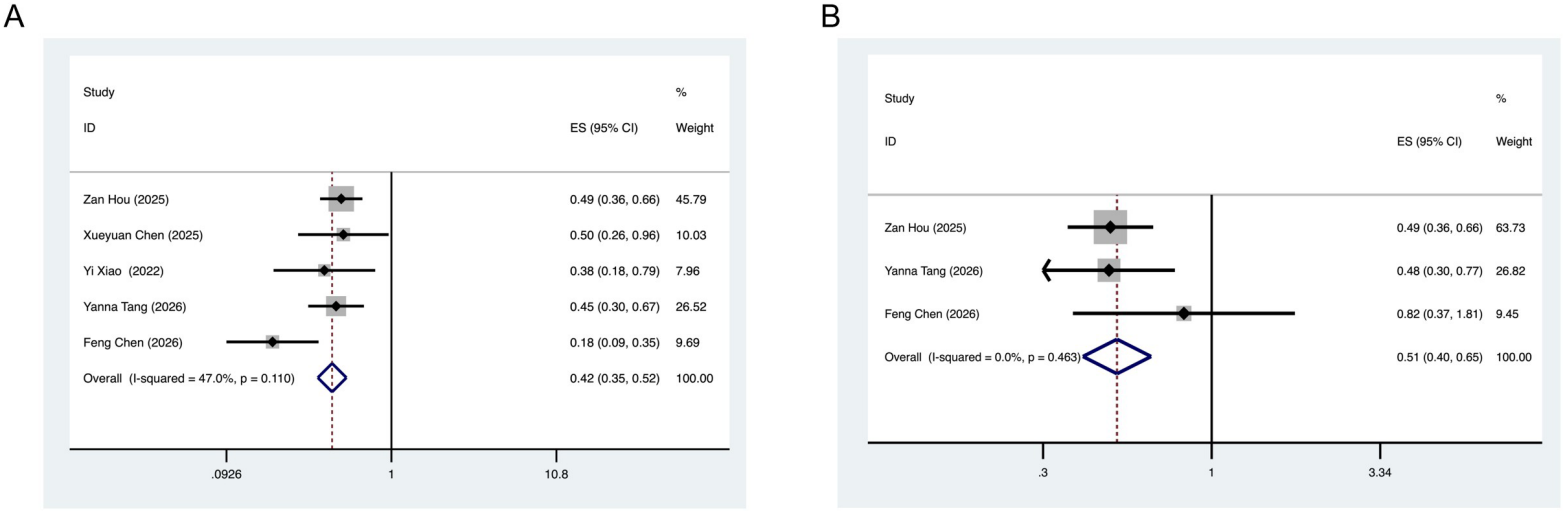
Updated meta-analysis: forest plot of hazard ratios for PFS **(A)** and OS **(B)** in patients with PLELC receiving first-line chemo-immunotherapy versus chemotherapy alone. PLELC, pulmonary lymphoepithelioma-like carcinoma;PFS, Progression-Free Survival;OS, overall survival.

Adding the Guangxi data kept statistical heterogeneity within acceptable limits for both PFS (I² = 47%, P = 0.110) and OS (I² = 0%, P = 0.463), justifying the continued use of a fixed-effects model. The combined survival benefits of chemoimmunotherapy remained highly robust. The updated pooled HR indicated a 58% reduction in the risk of disease progression (pooled HR = 0.42, 95% CI: 0.35–0.52, P < 0.001) and a 49% reduction in mortality risk (pooled HR = 0.51, 95% CI: 0.40–0.65, P < 0.001) (**Figure 7**, **Table 2**).

#### 3.4.2 Updated tumor response evidence (ORR and DCR)

**Figure 8:**
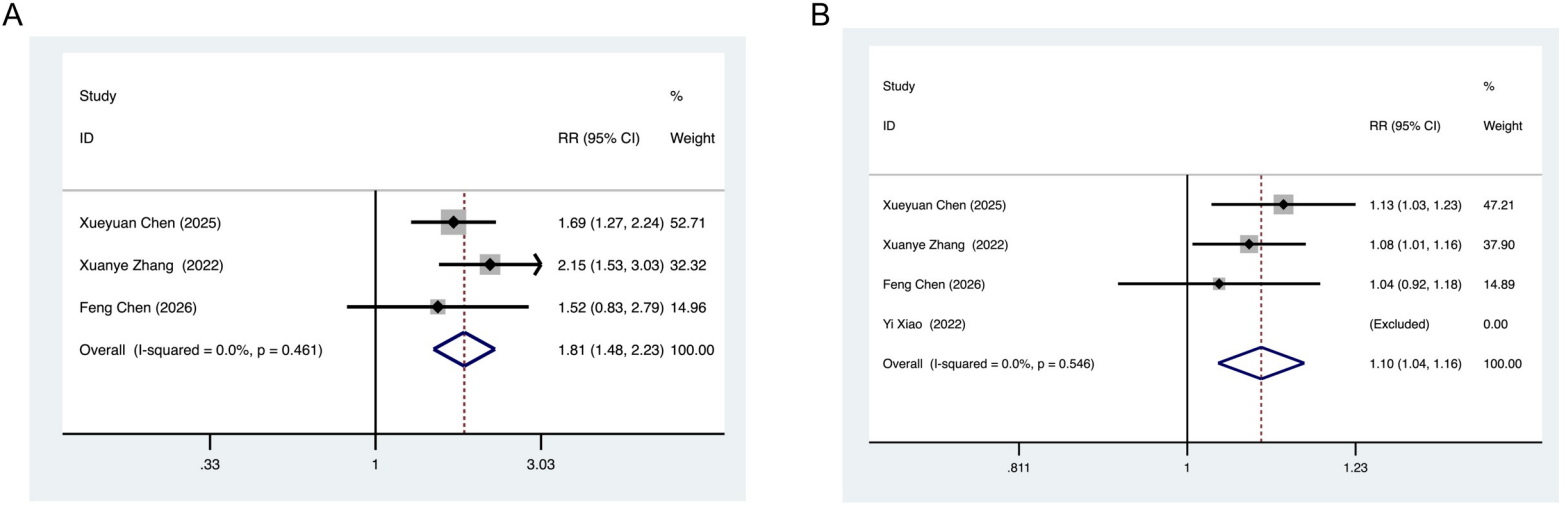
Updated meta-analysis: forest plot of relative risk for ORR **(A)** and DCR **(B)** in patients with PLELC receiving first-line chemo-immunotherapy versus chemotherapy alone. PLELC, pulmonary lymphoepithelioma-like carcinoma;ORR, Objective Response Rate; DCR, Disease Control Rate.

For the updated response metrics, we observed zero statistical heterogeneity across the combined cohorts for both ORR (I² = 0%, P = 0.461) and DCR (I² = 0%, P = 0.546). Applying a fixed-effects model, the updated synthesis confirmed that the IO+Chemo group consistently outperformed the chemotherapy-alone group in ORR (pooled RR = 1.81, 95% CI: 1.48–2.23, P < 0.001) and DCR (pooled RR = 1.10, 95% CI: 1.04–1.16, P = 0.001) (**Figure 8**, **Table 2**).

This low statistical heterogeneity suggests that the combination therapy delivers consistent clinical efficacy across the broader population, even when factoring in the complex baseline characteristics typical of real-world practice. An updated leave-one-out sensitivity analysis further confirmed that introducing these real-world effect sizes did not skew the overall evidence trajectory (**Supplementary Figure 9**). Finally, symmetrical funnel plots alongside non-significant Begg’s test results (all P > 0.05) suggested no overt publication bias, though statistical power remains limited (**Supplementary Figure 10**).

### 3.5 Exploratory analysis of anti-angiogenic combinations: efficacy and tolerability

**Figure 9:**
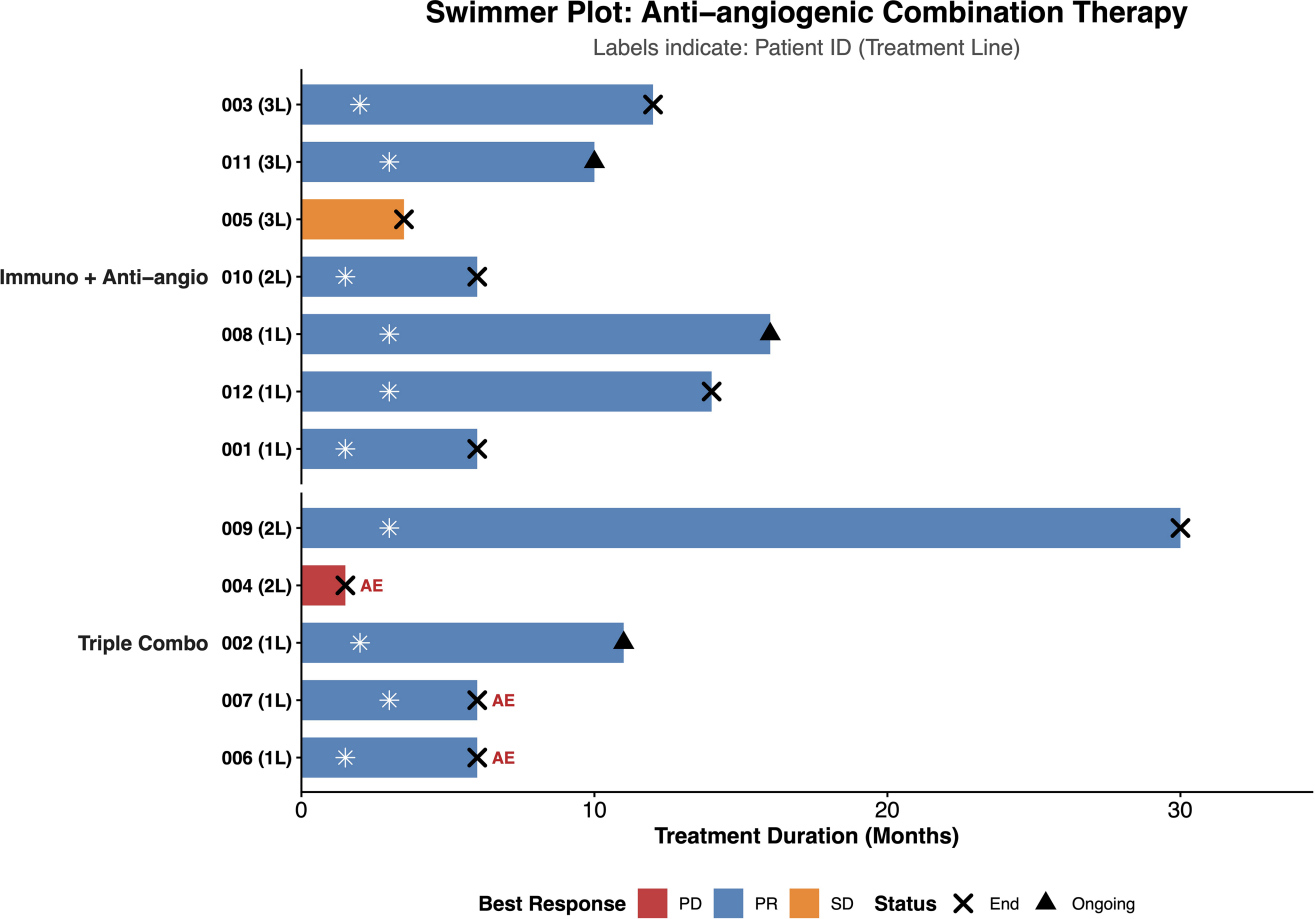
Swimmer plot of individual treatment exposure and response durability in the exploratory subgroup (IO plus Anti-angiogenic therapy ± Chemotherapy). AE, adverse event; Anti-angio, anti-angiogenic therapy; Chemo, chemotherapy; IO, immunotherapy; PD, progressive disease; PR, partial response; SD, stable disease.

We further evaluated a subset of 12 advanced PPLELC patients who received anti-angiogenic agents alongside immunotherapy, with or without chemotherapy. Seven patients underwent a doublet regimen (IO + anti-angiogenesis), while five received a triplet combination (IO + chemotherapy + anti-angiogenesis). Treatment lines varied, comprising six first-line, three second-line, and three third-line cases. Clinical assessments revealed an objective response rate (ORR) of 83.3% (10/12) and a disease control rate (DCR) of 91.7% (11/12), with best overall responses including 10 partial responses (PR), one stable disease (SD), and one progressive disease (PD). As depicted in the swimmer plot (**Figure 9**), most responders achieved a confirmed PR during their initial imaging scans between 1.5 and 3 months. Highlighting response durability, one patient on the second-line triplet regimen achieved 30 months of sustained disease control. At the data cutoff, three individuals remained in ongoing PR with continuous treatment exceeding 10 months (**Figure 9**). Tolerability profiles, however, differed noticeably between the regimens. All three documented cases of early treatment termination driven by adverse events (AEs) occurred within the triplet cohort, translating to a 60% discontinuation rate (3/5). In contrast, the doublet cohort experienced no AE-related dropouts (0% [0/7]).

## 4 Discussion

Advanced primary pulmonary lymphoepithelioma-like carcinoma (PPLELC) is an uncommon malignancy defined by strict geographic clustering (32, 33). Because of this localized incidence, securing cross-regional epidemiological data is critical to confirm the broad applicability of current immune-based treatments. To address this clinical gap, we engineered a dual-evidence framework—pairing a comprehensive systematic review with an IPTW-matched, multicenter real-world cohort. Utilizing an independent dataset from the highly endemic Guangxi region, our findings do more than merely reaffirm the benefits of fris-tline chemoimmunotherapy in shrinking target lesions and prolonging long-term survival; they establish the regimen’s cross-regional consistency and therapeutic robustness.Separately, the distinct tolerability profiles observed in our anti-angiogenic subgroup provide preliminary rationale for exploring “chemo-free” doublets in frail patient populations.

Our baseline meta-analysis of 606 patients demonstrated that adding immunotherapy to first-line chemotherapy significantly extends both PFS and OS, while boosting objective response rates without amplifying the overall toxicity burden (safety RR = 0.94). Building on this foundation, we injected the adjusted HRs from our Guangxi cohort directly into the meta-analysis model. The updated synthesis yielded highly stable survival benefits (PFS HR = 0.42, OS HR = 0.51) and lacked significant statistical heterogeneity. Crucially, these updated figures demonstrate that the chemoimmunotherapy regimen performs consistently well across the two primary endemic epicenters (Guangdong and Guangxi), largely dispelling concerns regarding regional efficacy bias.The pooled outcomes exhibited strong consistency across all included studies.Furthermore, leave-one-out sensitivity analyses verified the stability of the pooled data, demonstrating that no individual cohort unduly drove the final conclusions.Comparing our results horizontally with the landmark trials of traditional non-small cell lung cancer (NSCLC) helps to evaluate the clinical benefit more objectively. In the KEYNOTE-189 and KEYNOTE-407 studies for lung adenocarcinoma and squamous cell carcinoma, the PFS HR (approximately 0.48-0.62) and OS HR (approximately 0.56-0.71) of the first-line combination regimen are both slightly inferior to our updated effect sizes (PFS HR = 0.42, OS HR = 0.51) (34, 35). This difference in the depth of benefit likely stems from the unique EBV-driven etiology and the highly infiltrated “hot tumor” immune microenvironment of PLELC, which make it show a higher sensitivity to immune checkpoint blockade (36). These comparative data suggest that chemoimmunotherapy has good application prospects in advanced PLELC, and it deserves further validation in subsequent prospective studies.

Contrasting with the earlier studies in our meta-analysis, our real-world cohort features a substantially higher uptake of first-line immunotherapy. This shift reflects recent clinical evolution: as immune checkpoint inhibitors have become standard of care, physicians now tend to prioritize this potent combination for high-risk patients. It explains why the unadjusted chemoimmunotherapy group harbored a markedly heavier baseline tumor burden, notably in liver (29.2% vs. 0%) and bone metastases (33.3% vs. 5.9%). Despite these adverse factors, the regimen retained a pronounced PFS benefit following IPTW adjustment (adjusted HR = 0.12), validating its robust real-world efficacy against complex baseline conditions. While our updated meta-analysis confirmed a definitive OS advantage, the real-world cohort demonstrated only a numerical trend. We believe this lack of statistical significance stems from three interwoven clinical factors rather than a true absence of efficacy. First, extensive treatment crossover profoundly confounded the long-term outcomes: 68% of patients in the chemotherapy-alone group eventually received salvage immunotherapy upon progression. As Yang et al. previously established, second-line immune regimens yield substantial survival gains in advanced PPLELC (37); this subsequent benefit inevitably diluted the frontline OS differences in our cohort. Second, survival evaluations of immune checkpoint inhibitors are inherently subject to lag-time bias. The delayed onset of immune activation typically causes early survival curves to overlap before any distinct clinical benefit emerges. Finally, capturing the hallmark ‘long-tail effect’—the durable survival plateau characteristic of immunotherapy responders (38)—fundamentally requires years of observation. Given our current follow-up timeframe, the cohort simply may not have had adequate time to achieve definitive statistical separation, making extended clinical monitoring essential.Beyond treatment efficacy, our prognostic analyses identified baseline liver metastasis as a strong independent risk factor for shortened OS (adjusted HR = 4.96), a finding that directly aligns with real-world observations by Zhang et al. (30).Additionally, our exploratory subgroup analysis revealed that patients harboring baseline PD-L1 positivity (TPS ≥ 1%) experienced a significantly longer PFS than their PD-L1-negative counterparts (18.0 vs. 11.5 months, P = 0.049). This preliminary finding suggests a potential association between baseline PD-L1 positivity and improved PFS. However, given the severely limited sample size (n=23) and the extreme imbalance between the groups (only 5 negative cases), this observation lacks sufficient statistical power (39). Therefore, rather than establishing PD-L1 as a definitive predictive biomarker, these data serve strictly as a hypothesis-generating signal that requires rigorous validation in larger, well-powered prospective cohorts.

In our exploratory subgroup evaluating immunotherapy paired with anti-angiogenesis, patients achieved an 83.3% ORR alongside durable disease control. Mechanistically, inhibiting the VEGF/VEGFR axis does more than merely normalize aberrant tumor vasculature. It actively reprograms the immunosuppressive microenvironment by curtailing the infiltration of regulatory T cells (Tregs) and myeloid-derived suppressor cells (MDSCs), acting in potent synergy with immune checkpoint blockades (40, 41). Regarding safety, while we observed a noticeable numerical difference in AE-driven discontinuation rates (60% for the triplet regimen versus 0% for the chemo-free doublet), the extremely small sample size precludes any definitive conclusions. Nevertheless, this preliminary toxicity profile generates the hypothesis that for frail or elderly patients (ECOG PS ≥ 2) deemed unfit for conventional chemotherapy, a “chemo-free” dual-targeted approach might help maintain anti-tumor activity while potentially reducing the toxicity burden. We strictly caution that these are solely hypothesis-generating observations, and the exact clinical value of this de-escalation strategy fundamentally requires rigorous validation through larger, prospective cohorts.

This study still has some limitations. First, our meta-analysis ultimately included just four independent cohorts, highlighting the scarcity of robust clinical evidence for advanced PPLELC. As recommended by the Cochrane guidelines, reliable meta-regression requires at least 10 studies per continuous covariate. With only four cohorts available, performing formal meta-regression to explore the initial heterogeneity in objective response rate (ORR) would be statistically invalid and prone to false-positive findings. Instead, utilizing a leave-one-out sensitivity analysis to exclude the outlier served as the most methodologically sound alternative. However, the zero statistical heterogeneity observed for both PFS and OS (I²=0) suggests that the primary survival benefits remain stable. Second, within our real-world cohort, although we applied a strict IPTW model to balance observable baseline characteristics, the retrospective design means unmeasured confounders cannot be completely eliminated. For instance, classic channeling bias was present, as clinicians preferentially prescribed chemoimmunotherapy to patients with a heavier baseline metastatic burden. Additionally, due to historical disparities in regional clinical awareness, crucial biomarker data such as baseline EBV-DNA viral loads were largely omitted in routine practice.Third, the sample sizes for our exploratory subgroups were severely underpowered. The PD-L1 subgroup contained only 23 patients (with just 5 negative cases), and the anti-angiogenic combination subgroup included merely 12 patients. Therefore, these specific observations lack the statistical power to inform definitive clinical decisions and must be treated strictly as hypothesis-generating signals. Finally, the fundamental absence of prospective randomized data limits the definitive certainty of our conclusions, making high-quality, multicenter trials an urgent clinical priority.

## 5 Conclusion

This integrated systematic review and multicenter real-world cohort study suggests that first-line chemoimmunotherapy is associated with improved PFS and response outcomes compared with chemotherapy alone in advanced PPLELC. The updated pooled analysis supports a potential OS benefit, but these findings should be interpreted cautiously given the retrospective evidence base and residual confounding. Furthermore, exploratory observations indicate that “chemo-free” regimens incorporating anti-angiogenic agents may offer a tolerable alternative for frail patients. However, these hypothesis-generating signals require strict prospective validation prior to clinical application. Finally, considering the adverse prognostic impact of baseline liver metastasis and the potential utility of PD-L1 profiling, future management of PPLELC should prioritize biomarker-driven, personalized therapeutic strategies.

## Data Availability

The raw data supporting the conclusions of this article will be made available by the authors, without undue reservation.
