# Supplementary material for: Efficacy and safety of first-line chemoimmunotherapy versus chemotherapy alone for advanced pulmonary lymphoepithelioma-like carcinoma: a systematic review and real-world cohort study
Source: Front Immunol. 2026 Jul 1;17:1872713. doi: 10.3389/fimmu.2026.1872713 (PMC13369242; doi:10.3389/fimmu.2026.1872713)
Supplement: Supplementary file 1 [file Table1.docx]

Supplementary Material

| **Table of Contents** | | |
| --- | --- | --- |
| Title | Content | Page |
| Table S1 | Literature search strategy. | 2 |
| Table S2 | Assessment of study quality using the NOS. | 3-4 |
| Figure S1 | Forest plot of relative risk for ORR before exclusion of the outlier study in patients with PLELC receiving first-line chemo-immunotherapy versus chemotherapy alone. | 5 |
| Figure S2 | L’Abbé plot for heterogeneity assessment of ORR. | 5 |
| Figure S3 | Radial plot for heterogeneity assessment of ORR. | 6 |
| Figure S4 | Sensitivity analysis of ORR before exclusion of the outlier study. | 6 |
| Figure S5 | Sensitivity analysis of the enrolled studies. | 7 |
| Figure S6 | Funnel plots of the enrolled studies. | 8 |
| Figure S7 | Kernel density plot illustrating the overlap of Propensity Scores (PS) between the two treatment groups. | 9 |
| Figure S8 | Love plot of standardized mean differences (SMD) for baseline covariates before and after IPTW adjustment | 9 |
| Figure S9 | Updated meta-analysis: Sensitivity analysis of the enrolled studies. | 10 |
| Figure S10 | Updated meta-analysis: Funnel plots of the enrolled studies. | 11 |

**Table S1. Literature search strategy.**

| Search Strategy | | |
| --- | --- | --- |
| PubMed ：((((((LELC[Title/Abstract]) OR (lung lymphoepithelioma‐ like carcinoma[Title/Abstract])) OR (PLELC[Title/Abstract])) OR (Pulmonary lymphoepithelioma‐like carcinoma[Title/Abstract])) OR (Lymphoepithelioma-Like Carcinoma[Title/Abstract])) OR (pulmonary lymphoepithelioma like carcinoma[Title/Abstract])) AND (((("Immunotherapy"[Mesh]) OR ((((((((((((Immunotherapies[Title/Abstract]) OR (PD-1[Title/Abstract])) OR (PD-L1[Title/Abstract])) OR (CTLA-4[Title/Abstract])) OR (Ipilimumab[Title/Abstract])) OR (Atezolizumab[Title/Abstract])) OR (Durvalumab[Title/Abstract])) OR (Pembrolizumab[Title/Abstract])) OR (Adebrelimab[Title/Abstract])) OR (Serplulimab[Title/Abstract])) OR (Tiragolumab[Title/Abstract])) OR (nivolumab[Title/Abstract]))) OR (("Combined Modality Therapy"[Mesh]) OR ((((((((((Chemoimmunotherapy[Title/Abstract]) OR (Therapy, Combined Modality[Title/Abstract])) OR (Combined Modality Therapies[Title/Abstract])) OR (Modality Therapies, Combined[Title/Abstract])) OR (Modality Therapy, Combined[Title/Abstract])) OR (Therapies, Combined Modality[Title/Abstract])) OR (Multimodal Treatment[Title/Abstract])) OR (Multimodal Treatments[Title/Abstract])) OR (Treatment, Multimodal[Title/Abstract])) OR (Treatments, Multimodal[Title/Abstract])))) OR (("Drug Therapy"[Mesh]) OR (((((((Chemotherapy[Title/Abstract]) OR (Chemotherapies[Title/Abstract])) OR (Pharmacotherapy[Title/Abstract])) OR (Pharmacotherapies[Title/Abstract])) OR (Therapy, Drug[Title/Abstract])) OR (Drug Therapies[Title/Abstract])) OR (Therapies, Drug[Title/Abstract]))))  CNKI:(肺淋巴上皮瘤样癌 OR 肺淋巴上皮样癌 OR 原发性肺淋巴上皮瘤样癌 OR 原发性肺淋巴上皮样癌 OR 肺LELC OR PPLELC) and (化疗 OR 化学治疗OR免疫治疗OR 化疗联合免疫治疗 OR 化学免疫治疗 OR 免疫联合化疗 ) | | |
| Search Strategy in PubMed and CNKI | | |
| #1 | PubMed:  Search（Pulmonary lymphoepithelioma‐like carcinoma[Title/Abstract]) OR (pulmonary lymphoepithelioma like carcinoma[Title/Abstract]) OR (PLELC[Title/Abstract]) OR (Lymphoepithelioma-Like Carcinoma[Title/Abstract]) OR (LELC[Title/Abstract]) | |
| #2  #3  #4  #5  #6  #7  #8  #9  #10  #11  #12  #1  #2  #3 | Search: "Immunotherapy"[Mesh]  Search (Immunotherapies[Title/Abstract]) OR (PD-1[Title/Abstract]) OR (PD-L1[Title/Abstract]) OR (CTLA-4[Title/Abstract]) OR (Ipilimumab[Title/Abstract]) OR (Atezolizumab[Title/Abstract]) OR (Durvalumab[Title/Abstract]) OR (Pembrolizumab[Title/Abstract]) OR (Adebrelimab[Title/Abstract]) OR (Serplulimab[Title/Abstract]) OR (Tiragolumab[Title/Abstract]) OR (nivolumab[Title/Abstract])  #2 OR #3  Search: "Drug Therapy"[Mesh]  Search (Drug Therapy[Title/Abstract]) OR (Chemotherapy[Title/Abstract]) OR (Chemotherapies[Title/Abstract])) OR (Pharmacotherapy[Title/Abstract])OR(Pharmacotherapies[Title/Abstract]) OR (Therapy, Drug[Title/Abstract]) OR (Drug Therapies[Title/Abstract]) OR (Therapies, Drug[Title/Abstract])   #5OR #6  Search: "Combined Modality Therapy"[Mesh]  Search (Chemoimmunotherapy[Title/Abstract]) OR (Therapy, Combined Modality[Title/Abstract]) OR (Combined Modality Therapies[Title/Abstract]) OR(Modality Therapies, Combined[Title/Abstract]) OR (Modality Therapy, Combined[Title/Abstract]) OR (Therapies, Combined Modality[Title/Abstract])OR(Multimodal Treatment[Title/Abstract]) OR (Multimodal Treatments[Title/Abstract]) OR (Treatment, Multimodal[Title/Abstract]) OR (Treatments, Multimodal[Title/Abstract])  #8OR #9  #4OR #7 OR #10  #1AND #11  CNKI:  Search (肺淋巴上皮瘤样癌 OR 肺淋巴上皮样癌 OR 原发性肺淋巴上皮瘤样癌 OR 原发性肺淋巴上皮样癌 OR 肺LELC OR PPLELC)  Search (化疗 OR 化学治疗 OR 免疫治疗OR化疗联合免疫治疗 OR 化学免疫治疗 OR 免疫联合化疗)  #1AND #2 | |
|  | |  |

**Supplementary Table 2.** **Assessment of study quality using the NOS**

| Study | Representativeness of the exposed cohort | Selection of the non-exposed cohort | Ascertainment of exposure | Outcomes of interest was not present at the start of the study | Comparability of cohorts on the basis of the design or analysis | Assessment of outcome | Follow-up was long | Adequacy of follow-up of cohorts | Total quality score |
| --- | --- | --- | --- | --- | --- | --- | --- | --- | --- |
| Zan Hou 2025 | 1 | 1 | 1 | 1 | 0 | 1 | 1 | 0 | 6 |
| Xueyuan Chen 2025 | 1 | 1 | 1 | 1 | 2 | 1 | 1 | 0 | 8 |
| Xiongwen Yang 2025 | 1 | 1 | 1 | 1 | 2 | 1 | 1 | 0 | 8 |
| Lan-Lan Pang 2023 | 1 | 1 | 1 | 1 | 2 | 1 | 1 | 0 | 8 |
| Yi Xiao 2022 | 1 | 1 | 1 | 1 | 2 | 1 | 0 | 0 | 7 |
| Xuanye Zhang 2022 | 1 | 1 | 1 | 1 | 2 | 1 | 1 | 1 | 9 |

Abbreviations: NOS, Newcastle–Ottawa quality assessment scale

Supplementary Table S3. Distribution of Specific Pharmacological Agents in the Real-World Cohort

| **Treatment Characteristics** | **Total Cohort**  (N = 65)^1^ | **Chemotherapy Alone**  N = 17^1^ | **Chemoimmunotherapy**  N = 48^1^ |
| --- | --- | --- | --- |
| **Platinum-based Agents** |  |  |  |
| Carboplatin | 41 (63.1%) | 10 (58.8%) | 31 (64.6%) |
| Cisplatin | 14 (21.5%) | 6 (35.3%) | 8 (16.6%) |
| Nedaplatin / Others | 10 (15.4%) | 1 (5.9%) | 9 (18.8%) |
| **Non-platinum Agents in Doublet** |  |  |  |
| Docetaxel | 10 (15.4%) | 6 (35.2%) | 4 (8.3%) |
| Gemcitabine | 5 (7.7%) | 0 (0%) | 5 (10.4%) |
| Paclitaxel | 44 (67.7%) | 8 (47.1%) | 36 (75%) |
| Pemetrexed/ Others | 5 (7.7%) | 2 (11.8%) | 3 (6.3%) |
| **PD-1/PD-L1 Inhibitors** |  |  |  |
| N/A | 17 (26.2%) | 17 (100%) | 0 (0%) |
| Others | 2 (3.1%) | 0 (0%) | 2 (4.2%) |
| Pembrolizumab | 3 (4.6%) | 0 (0%) | 3 (6.2%) |
| Sintilimab | 9 (13.8%) | 0 (0%) | 9 (18.8%) |
| Tislelizumab | 29 (44.6%) | 0 (0%) | 29 (60.4%) |
| Camrelizumab | 5 (7.7%) | 0 (0%) | 5 (10.4%) |
| ^1^n (%) | | | |

Figure S1：Forest plot of relative risk for ORR before exclusion of the outlier study in patients with PLELC receiving first-line chemo-immunotherapy versus chemotherapy alone. ORR, objective response rate.

Figure S2：L’Abbé plot for heterogeneity assessment of ORR. ORR, objective response rate.

Figure S3：Radial plot for heterogeneity assessment of ORR. ORR, objective response rate.


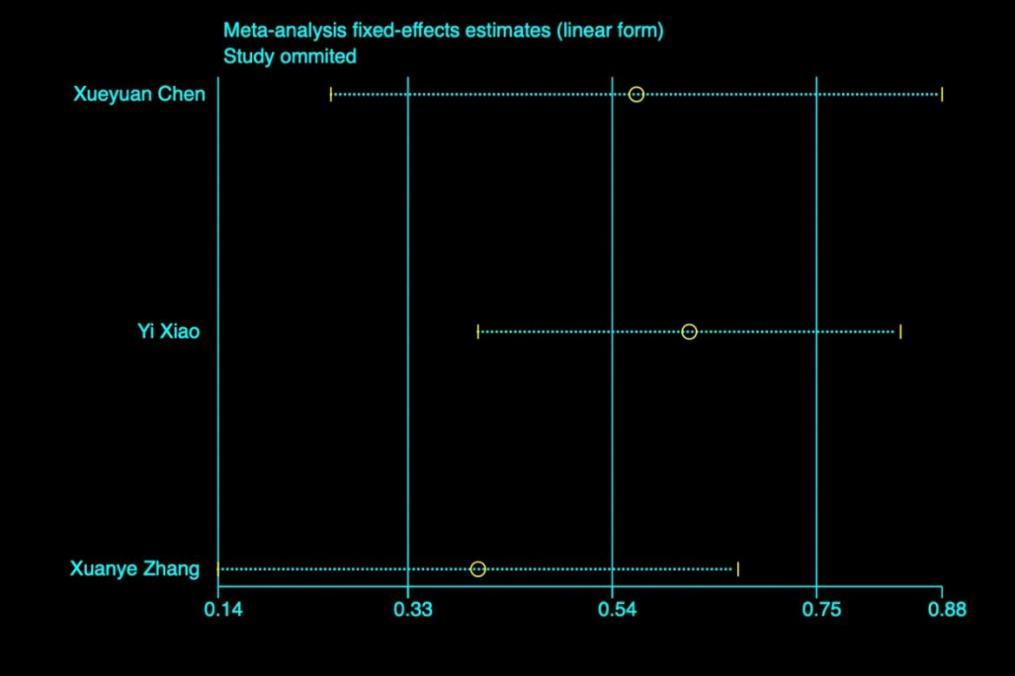


Figure S4：Sensitivity analysis of ORR before exclusion of the outlier study. ORR, objective response rate.


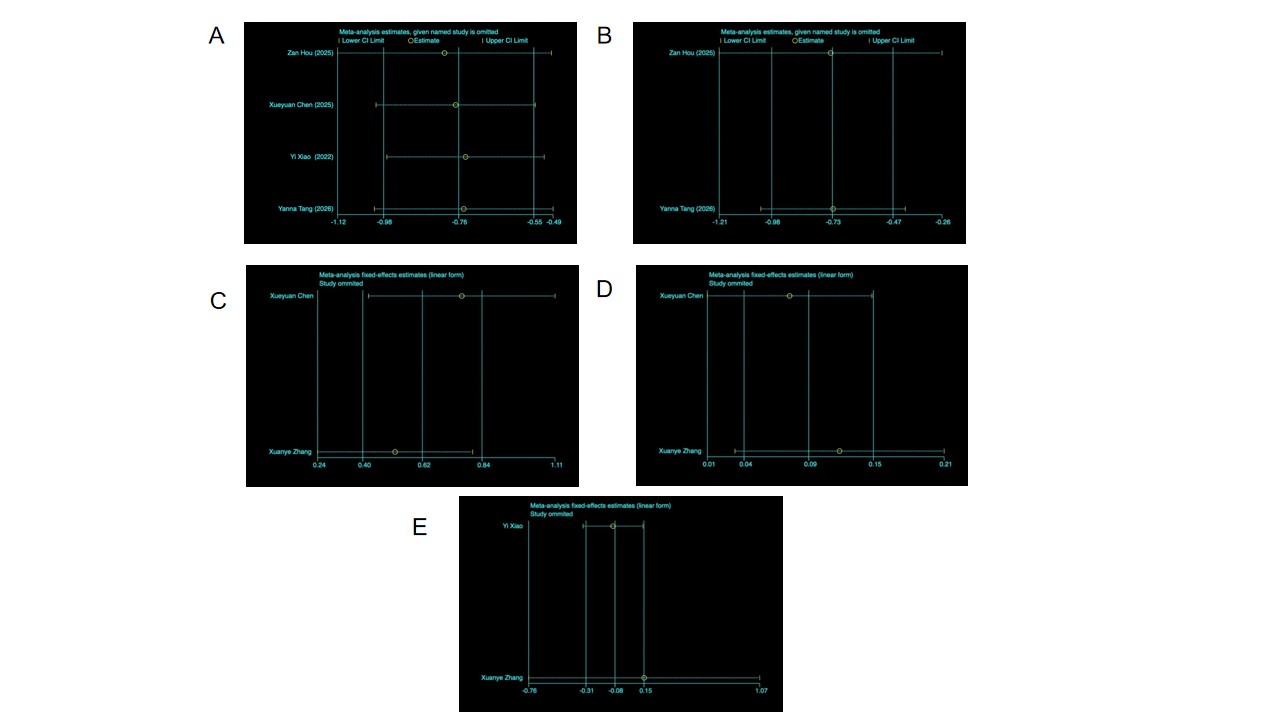


Figure S5. Sensitivity analysis of the enrolled studies. (A) Chemo‑immunotherapy vs Chemotherapy and PFS. (B) P Chemo‑immunotherapy vs Chemotherapy and OS.(C)Chemo‑immunotherapy vs Chemotherapy and ORR.(D)Chemo‑immunotherapy vs Chemotherapy and DCR .(E) Chemo‑immunotherapy vs Chemotherapy and grade 3/4 TRAE.OS, overall survival; PFS, progression-free survival; DCR, disease control rate; ORR, objective response rate; TRAE: Treatment-Related Adverse Events


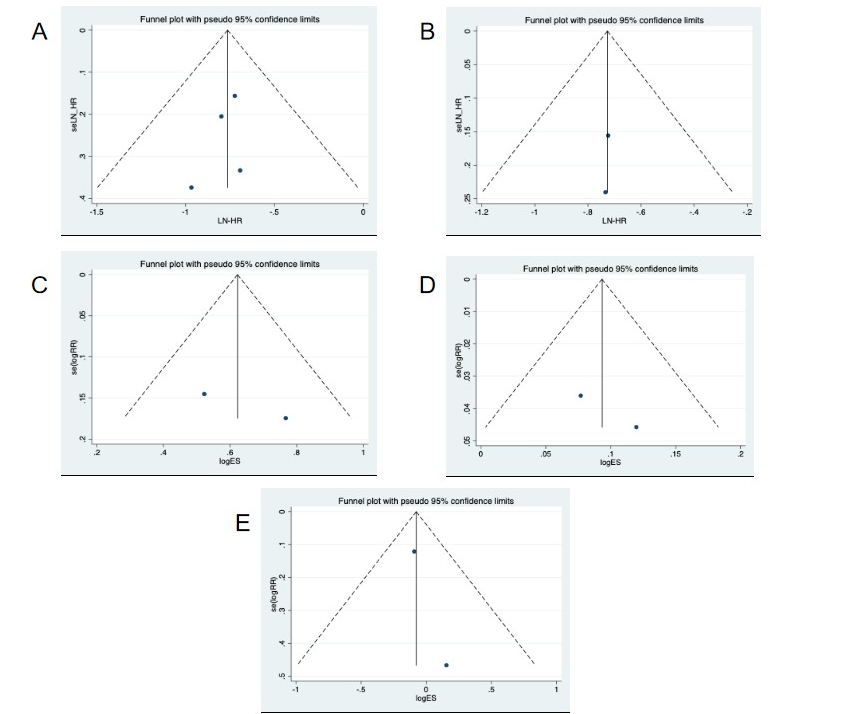


Figure S6. Funnel plots of the enrolled studies. (A) PFS in Chemo‑immunotherapy vs Chemotherapy (B) OS in Chemo‑immunotherapy vs Chemotherapy group. OS, overall survival; PFS, progression-free survival. (C) ORR in Chemo‑immunotherapy vs Chemotherapy andORR(D)DCR in Chemo‑immunotherapy vs Chemotherapy(E)Grade 3/4 TRAE in Chemo‑immunotherapy vs Chemotherapy.OS, overall survival; PFS, progression-free survival; DCR, disease control rate; ORR, objective response rate; TRAE: Treatment-Related Adverse Events.

Figure S7. Kernel density plot illustrating the overlap of Propensity Scores (PS) between the two treatment groups.


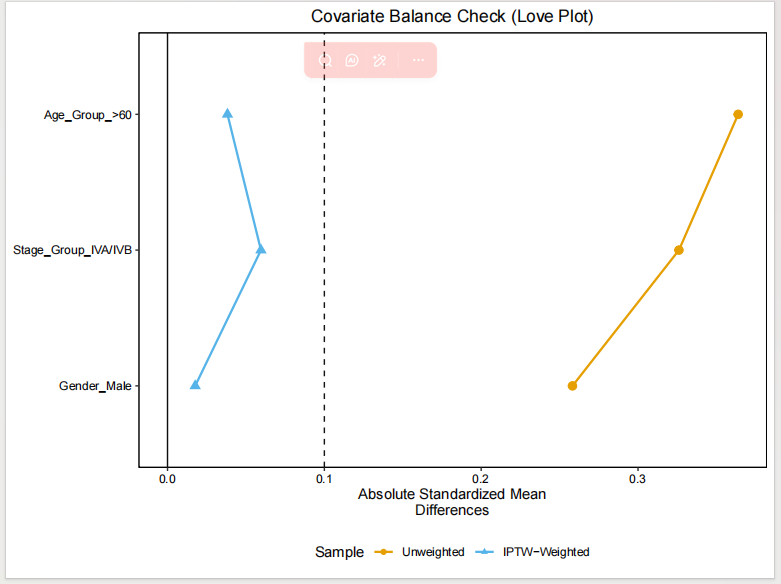


Figure S8. Love plot of standardized mean differences (SMD) for baseline covariates before and after IPTW adjustment


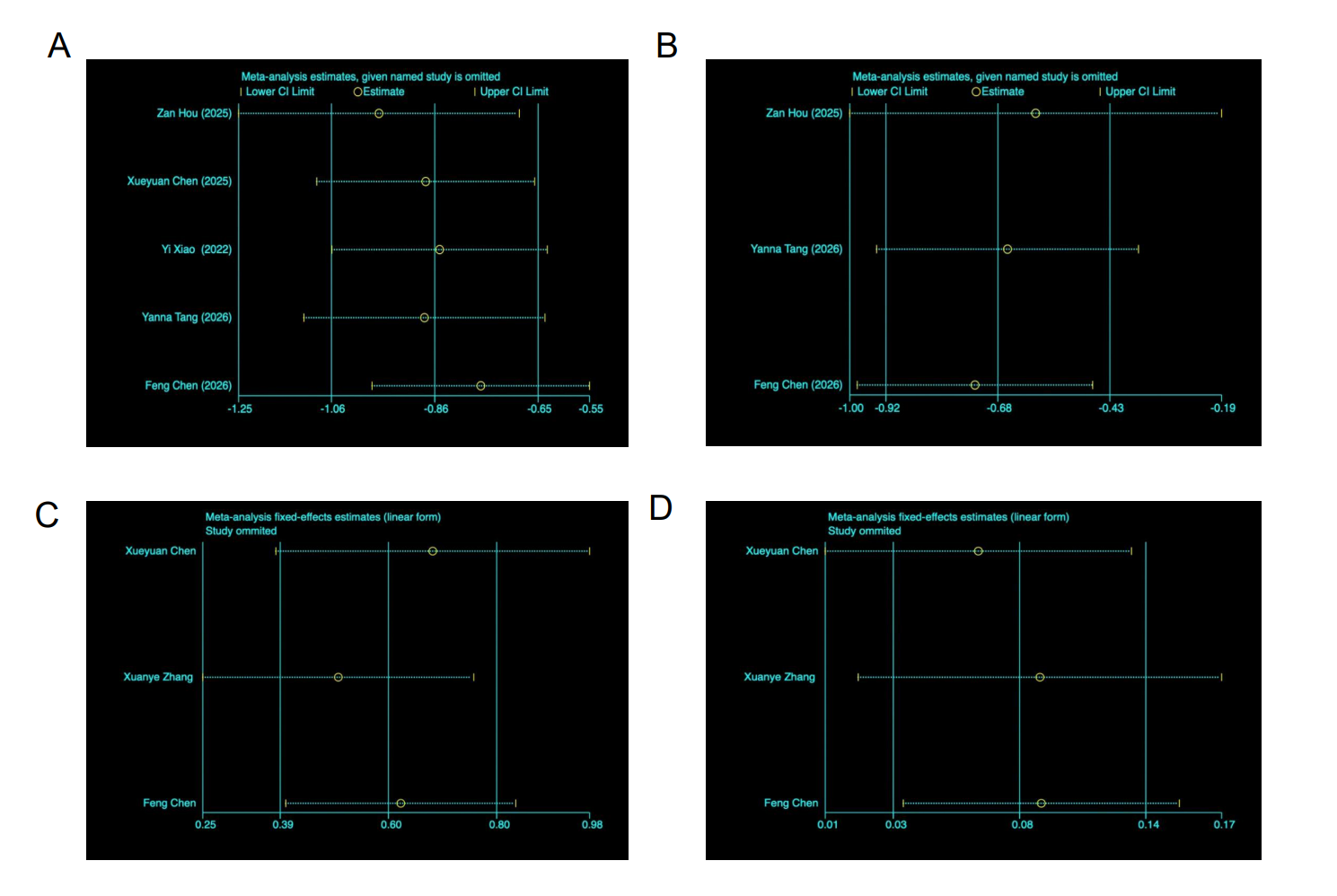


Figure S9. Updated meta-analysis: Sensitivity analysis of the enrolled studies. (A) Chemo‑immunotherapy vs Chemotherapy and PFS. (B) P Chemo‑immunotherapy vs Chemotherapy and OS.（C）Chemo‑immunotherapy vs Chemotherapy andORR（D）Chemo‑immunotherapy vs Chemotherapy and DCR.OS, overall survival; PFS, progression-free survival; DCR, disease control rate; ORR, objective response rate.


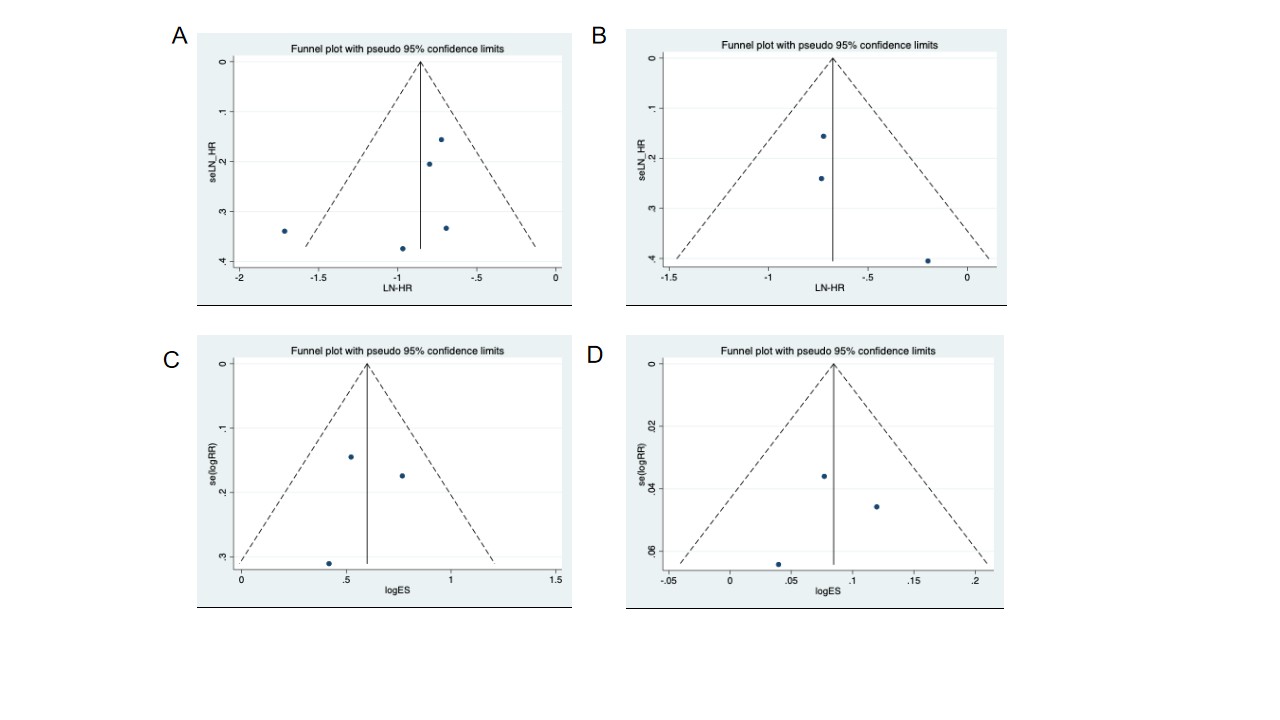


Figure S10. Updated meta-analysis: Funnel plots of the enrolled studies. (A) PFS in Chemo‑immunotherapy vs Chemotherapy (B) OS in Chemo‑immunotherapy vs Chemotherapy group.. OS, overall survival; PFS, progression-free survival. (C)ORR in Chemo‑immunotherapy vs Chemotherapy and ORR. (D)DCR in Chemo‑immunotherapy vs Chemotherapy. OS, overall survival; PFS, progression-free survival; DCR, disease control rate; ORR, objective response rate.
